# Polycystic ovary syndrome and 25-hydroxyvitamin D: A bidirectional two-sample Mendelian randomization study

**DOI:** 10.3389/fendo.2023.1110341

**Published:** 2023-03-09

**Authors:** Nana Zhang, Yan Liao, Hongyu Zhao, Tong Chen, Fan Jia, Yue Yu, Shiqin Zhu, Chaoying Wang, Wufan Zhang, Xinmin Liu

**Affiliations:** ^1^ Graduate School, Beijing University of Chinese Medicine, Beijing, China; ^2^ Guang’anmen Hospital, China Academy of Chinese Medical Sciences, Beijing, China

**Keywords:** polycystic ovary syndrome, 25-hydroxyvitamin D, Mendelian randomization, obesity, insulin resistance, causal inference, genetic epidemiology

## Abstract

**Background:**

Accumulating observational studies have indicated that vitamin D deficiency (serum 25-hydroxyvitamin D (25OHD) < 50 nmol/L) is common in women with polycystic ovary syndrome (PCOS). However, the direction and causal nature remain unclear. In this study, we aimed to investigate the causal association between PCOS and 25OHD.

**Methods:**

A bidirectional two-sample Mendelian randomization (MR) study was used to evaluate the causal association between PCOS and 25OHD. From the publicly available European-lineage genome-wide association studies (GWAS) summary statistics for PCOS (4,890 cases of PCOS and 20,405 controls) and 25OHD (n = 417,580), we selected 11 and 102 single nucleotide polymorphisms (SNPs) as instrumental variables (IVs), respectively. In univariate MR (uvMR) analysis, inverse-variance weighted (IVW) method was employed in the primary MR analysis and multiple sensitivity analyses were implemented. Additionally, a multivariable MR (mvMR) design was carried to adjust for obesity and insulin resistance (IR) as well.

**Results:**

UvMR demonstrated that genetically determined PCOS was negatively associated with 25OHD level (IVW Beta: -0.02, *P* = 0.008). However, mvMR found the causal effect disappeared when adjusting the influence of obesity and IR. Both uvMR and mvMR analysis didn’t support the causal effect of 25OHD deficiency on risk of PCOS (IVW *OR*: 0.86, 95% *CI*: 0.66 ~ 1.12, *P* = 0.280).

**Conclusion:**

Our findings highlighted that the casual effect of PCOS on 25OHD deficiency might be mediated by obesity and IR, and failed to find substantial causal effect of 25OHD deficiency on risk of PCOS. Further observational studies and clinical trials are necessary.

## 1 Introduction

Polycystic ovary syndrome (PCOS) is a common and complex endocrinopathy affecting approximately 4%-21% of women of reproductive age around the world (1). Characterized mainly by ovulation disorder, clinical and/or biological hyperandrogenism and/or an ultrasound aspect of polycystic ovaries (1, 2), it has been confirmed that PCOS is associated with certain metabolic disorders, such as obesity, insulin resistance(IR) and sometimes even type 2 diabetes mellitus (T2DM) (3).

Vitamin D, as an easy to supplementation and fat-soluble vitamin, has aroused great interest in the link with PCOS in recent decades. 25-hydroxyvitamin D (25OHD) is the main circulating form to evaluate the status of vitamin D. Accumulating observational studies have indicated that vitamin D deficiency [serum 25OHD < 50 nmol/L (4)] is common in women with PCOS. Convincing evidence has shown that 25OHD deficiency is associated with obesity (5, 6) and IR (7). Metabolic disorders, such as obesity and IR, has been proposed as the possible confounding factors between PCOS and 25OHD deficiency (8). In fact, due to confounding factors and reverse causation, the real relationship between PCOS and 25OHD remains unclear.

Mendelian randomization (MR) can strengthen inferring causality and give a robust estimation between a risk factor and an outcome of interest (9). Compared with observational studies, MR, in particular, multivariable MR (mvMR), uses single nucleotide polymorphisms (SNPs) identified from the genome-wide association study (GWAS) as instrumental variables (IVs) and is less susceptible to confounding, as genetic variants are randomly allocated at conception (10, 11). Therefore, we integrated the univariable MR (uvMR), mvMR and bidirectional MR approaches to investigate the possible causal association between PCOS and 25OHD.

## 2 Methods

### 2.1 Study design

**Figure 1:**
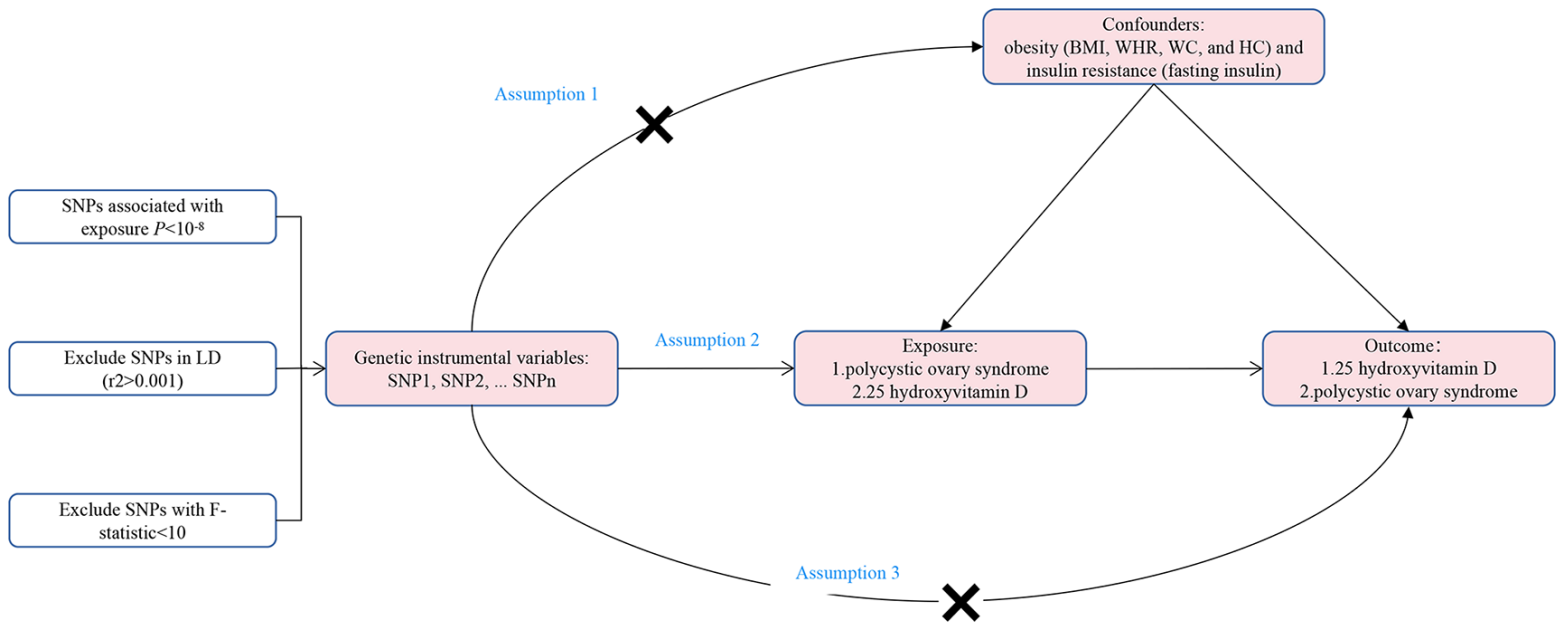
The framework of the bidirectional two-sample Mendelian randomization study between polycystic ovary syndrome and 25-hydroxyvitamin D. SNP, single nucleotide polymorphism; BMI, body mass index; WHR, waist-to-hip ratio; WC, waist circumference; HC, hip circumference; LD, linkage disequilibrium.

The bidirectional two-sample MR study was conducted in the framework shown in **Figure 1**. In order to have a valid interpretation for the MR analysis, it is necessary that the following three assumptions (10) hold: Assumption 1, IVs are robustly correlated with the exposure; Assumption 2, IVs are independent of any confounding factors between the relationship of exposure and outcome; Assumption 3, IVs influence outcome only *via* exposure. In this study, SNPs were employed as IVs to perform bidirectional two-sample MR to explore the causal association between PCOS and 25OHD.

### 2.2 Equations data sources

#### 2.2.1 GWAS summary statistics of PCOS

The GWAS summary statistics of PCOS were obtained from the latest and largest published GWAS meta-analysis, including 4,890 patients with PCOS and 20,405 health controls of European ancestry, and were adjusted for age (12) (**Supplemental Material S1**). Diagnosis of PCOS was based on National Institutes of Health criteria (13), Rotterdam criteria (2), or self-report questionnaire (14).

#### 2.2.2 GWAS summary statistics of 25OHD

The GWAS summary statistics of 25OHD were obtained from 417,580 European UK Biobank participants with available serum 25OHD levels (6), and adjusted for age at assessment, sex, assessment month, assessment center, first four ancestry principal components, genotyping batch, supplement intake (variable with four levels, namely: “no information”, “never taken”, “other supplements”, “25OHD supplements”) (6) (**Supplemental Material S1**).

### 2.3 Ethical approval

Our MR study was performed using publicly published studies or shared datasets, which had acquired ethical approval and informed consent. No additional ethics statement or consent was required.

### 2.4 Instrumental variable selection

SNPs were chosen as IVs, which sanctified the genome-wide significance threshold of *p* < 5 × 10^–8^, and none of which surpassed the limited value (r^2^ < 0.001 within a clumping window of 10,000 kb) in linkage disequilibrium (LD) analysis (15). Palindromic SNPs with regardless of allele frequency were removed from the analyses (15). Only the SNPs that existed in the GWAS summary statistics of outcome were included as IVs, and the proxy SNPs were not included in the analysis (16, 17). Each SNPs’ power was evaluated by the *F* statistics (*F* = Beta^2^/SE^2^) (18). Finally, SNPs with less statistical power would be removed (*F* statistics<10). The proportion of variance of the exposure explained by SNPs was evaluated by R^2^. R^2^ of each SNP was calculated by the following formula: 2 × EAF × (1-EAF) × Beta^2^, and summed them up to calculate the overall R^2^, where EAF represents the effect allele frequency of the SNP (19).

Eventually, 11 SNPs for PCOS and 102 SNPs for 25OHD were separately extracted from the GWAS summary statistics. The *F* statistics of all selected SNPs ranged separately from 30.84 to 57.66 and 30.07 to 1,468.55, demonstrating that all selected SNPs had sufficient validity. Moreover, the total variance explained by the genetic instruments was 6.23% and 2.71%, respectively. The detailed information of all selected SNPs of PCOS and 25OHD was presented in **Supplemental Material S2-S3**.

### 2.5 Mendelian randomization estimates

Fixed-effect inverse-variance weighted (IVW) method was conducted as the primary methods for uvMR estimates (20). The IVW method requires all instrumental variants to be valid, and will return an unbiased estimate if the horizontal pleiotropy is balanced (15). MR-Egger, weighted median, MR-pleiotropy residual sum and outlier (MR-PRESSO) (21) were further employed to control for horizontal pleiotropy as complementary methods (20). MR-Egger regression has a lower statistical power with a wide range of causality estimates (22). It adapts the IVW analysis by allowing a non-zero intercept, allowing the net-horizontal pleiotropic effect across all SNPs to be unbalanced, or directional (15, 22). If more than 50% of the weight derived from effective IVs, the weighted median method may yield a more robust estimate of causality (23). MR-PRESSO considered the horizontal pleiotropy and could detect and correct for outlier SNPs reflecting pleiotropic biases (21). In addition, leave-one-out sensitivity analysis, Cochran’s Q statistic and MR-Egger intercept test (22, 24) were also used to test for heterogeneity and pleiotropy. MvMR was adopted to estimate the direct effect of exposure on outcome independent of important confounders including obesity and IR. Body mass index (BMI), waist-to-hip ratio (WHR), waist circumference (WC) and hip circumference (HC) were selected as indicators to access obesity. IR was represented by fasting insulin. The GWAS summary statistics of BMI, WHR, WC, HC and fasting insulin were all from the publicly available IEU Open GWAS Project database (https://gwas.mrcieu.ac.uk/ ), for which details were shown in **Supplemental Material S1**.

MR analyses were performed using the packages “TwoSampleMR” (15) (version 0.5.6) and “MRPRESSO” (version 1.0) through R Software (version 4.1.2). Statistical significance was set at *P* < 0.05.

## 3 Results

### 3.1 Causal association of PCOS on 25OHD *via* forward MR

**Figure 2:**
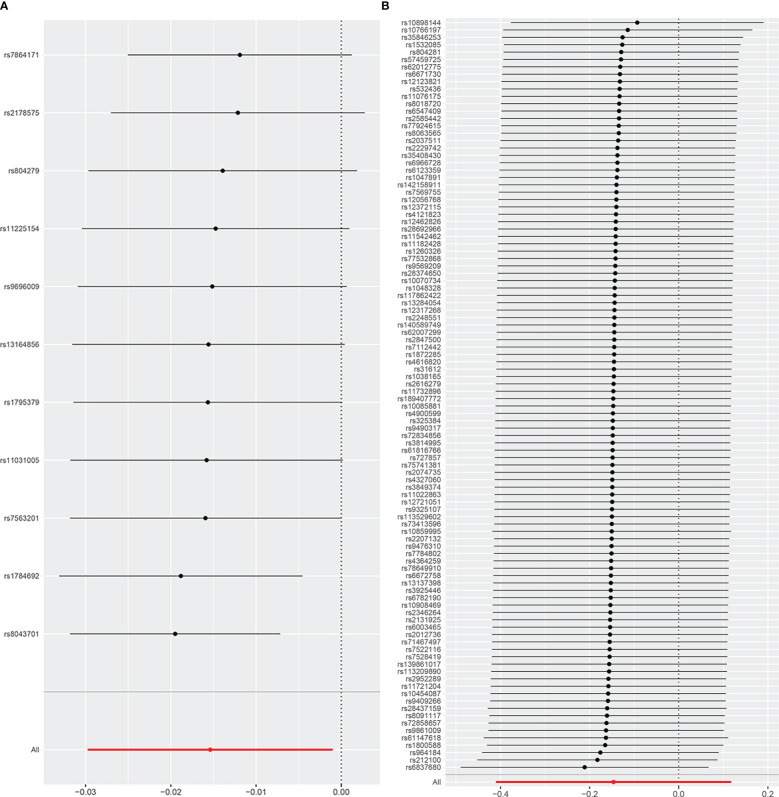
The leave-one-out analysis plot **(A)** Leave-one-out plot for PCOS on 25OHD. **(B)** Leave-one-out plot for 25OHD on PCOS.

The results of the uvMR analyses were shown in **Table 1**. The IVW method showed that genetically determined concentrations of PCOS were negatively associated with 25OHD. In the main IVW analysis, per standard deviation increase in PCOS was associated with decrease in 25OHD concentration of 0.02 nmol L^−1^ (*OR* = 0.98, 95% *CI* = 0.97~1.00, *P* = 0.008). All other MR estimates were consistent with the direction of the main IVW estimate (*P* > 0.05), and no outlier SNPs were observed in the MR-PRESSO analysis. However, there were some SNPs crossing the zero line in leave-one-out sensitivity analysis, which suggested potential heterogeneity (**Figure 2A**). Cochran’s Q statistics showed there remained little evidence of heterogeneity among SNPs of PCOS. No pleiotropy was detected (MR-Egger intercept = 0.070, *P* = 0.710).

**Table 1:** Results of causal associations between PCOS and 25OHD.

Exposure	Outcome	Method	N of SNPs	Beta	SE	OR (95%CI)	*P*	Q stastistic	*P*-heterogeneity	*P*-intercept
PCOS	25OHD	IVW	11	-0.02	0.01	0.98(0.97∼1.00)	0.008			
		MR Egger	11	<-0.001	0.04	1.00(0.93∼1.08)	0.970	15.90	0.07	0.71
		Weighted median	11	-0.01	0.01	0.99(0.97∼1.00)	0.110			
		MR-PRESSO	11	-0.02	0.01	0.98(0.97∼1.00)	0.062			
25OHD	PCOS	IVW	102	-0.15	0.13	0.86(0.66∼1.12)	0.276			
		MR Egger	102	-0.03	0.22	0.97(0.64∼1.48)	0.886	97.69	0.55	0.49
		Weighted median	102	-0.37	0.21	0.69(0.45∼1.05)	0.082			
		MR-PRESSO	102	-0.15	0.13	0.86(0.67∼1.12)	0.272			

PCOS, polycystic ovary syndrome; 25OHD, 25-Hydroxyvitamin D; IVW, inverse-variance weighted; MR-Egger, Mendelian randomization Egger; SNP, single nucleotide polymorphism; SE, standard error; OR, odds ratio; 95% CI, 95% confidence interval; P-heterogeneity, p-value of heterogeneity test from Cochrane's Q value; P-intercept, p-value of pleiotropy test from MR-Egger intercept.

**Figure 3:**
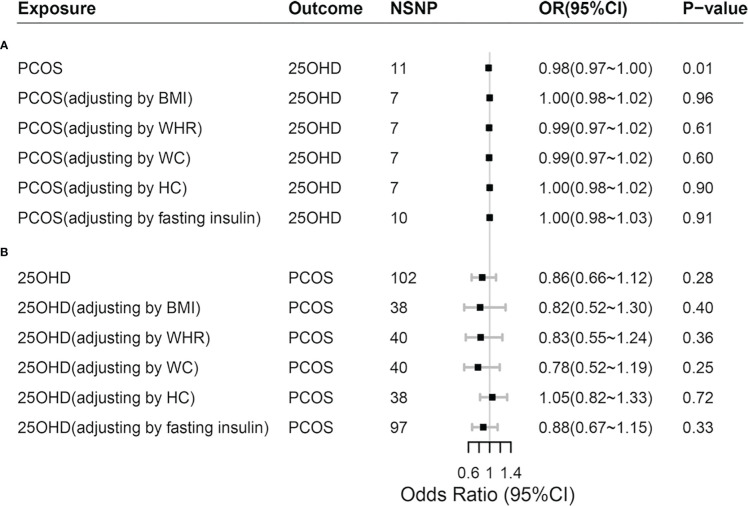
Comparisons of Mendelian randomization results. **(A)** Comparisons of Mendelian randomization results for PCOS on 25OHD; **(B)** Comparisons of Mendelian randomization results for 25OHD on PCOS. PCOS, polycystic ovary syndrome; 25OHD, 25-Hydroxyvitamin D; OR, odds ratio; 95% CI, 95% confidence interval; BMI, body mass index; WHR, waist-to-hip ratio; WC, waist circumference; HC, hip circumference.

Adjusting the influence of confounders including obesity (BMI, WHR, WC and HC) and IR (represented by fasting insulin) in mvMR analysis, we found that it was disappeared for the causal relationship between genetically predicted PCOS and 25OHD (*P* > 0.05) (**Figure 3**; **Supplemental Material S4**). In addition, we found that BMI decreased 25OHD concentration (Beta: -0.09, *P* < 0.001), which was in keeping with previous MR study (6, 25).

### 3.2 Causal association of 25OHD on PCOS *via* reverse MR

As shown in **Table 1**, the IVW method showed *OR* = 0.86, 95% *CI* = 0.66~1.12, *P* = 0.280, and the other uvMR methods also obtained generally consistent results (*P* > 0.05) with the main method in the opposite direction. There was no evidence suggested the possible causal relationship between genetically predicted 25OHD and PCOS. Leave-one-out sensitivity analysis (**Figure 2B**) did not identify any leverage points with high influence, suggesting the stability and reliability of MR results. Cochran’s Q statistics showed there was no heterogeneity among SNPs of 25OHD. The MR-Egger intercept test demonstrated no evidence of directional pleiotropy (*P* > 0.05). Besides, no outlier SNPs were observed in the MR-PRESSO analysis.

Adjusting the influence of confounders including obesity and IR in mvMR analysis, we still found no causal relationship between genetically predicted 25OHD and PCOS (*P* > 0.05), which was consistent with the results of univariable MR (**Figure 3**; **Supplemental Material S4**). In addition, when 25OHD and BMI were assessed together using mvMR method, we found that genetically predicted BMI increased the risk of PCOS (*OR* = 2.98, 95% *CI* = 2.12~4.19, *P* < 0.001), so did fasting insulin (*OR* = 2.30, 95% *CI*=1.02~5.15, *P* = 0.044).

## 4 Discussion

This study explored the relationships between PCOS and 25OHD using a bidirectional two-sample MR design for the first time, which adopted the strong IVs from the largest GWAS of respective phenotypes in European populations. Our study found that genetically predicted PCOS was weakly inversely associated with deficiency of 25OHD. However, the casual association may be unstable and would disappear after correcting the influence of obesity and IR. The effects of PCOS on 25OHD might be accounted for obesity and IR. Meanwhile, our study didn’t support the causal effect of 25OHD on PCOS.

Previous observational studies have found that there seems to be a link between PCOS and 25OHD, while the causality is still not yet fully clear. Some studies reported that PCOS was a risk factor for vitamin D insufficiency (26–30). Recently, a retrospective cross-sectional study involving Chinese with PCOS also discovered that the low level of 25OHD was prevalent in PCOS women (31). On the contrary, a systematic review and meta-analysis found that lower concentrations of serum 25OHD lead to a greater risk of developing PCOS (32). Besides, Panidis D et al.’s study grouped the patients with PCOS and controls according to BMI (33). They found that increased BMI had a significant negative effect on 25OHD, but didn’t support the association between PCOS on 25OHD (33). Some studies showed convincing data that low 25OHD levels were associated with obesity and IR, but not with PCOS per se (26, 32, 33), which was consistent with our outcome.

The real relationship between PCOS and 25OHD might be interpreted as follows: An increasing body of clinical evidence (27, 28, 34, 35) suggested that insufficient 25OHD were associated with hyperandrogenism, metabolic syndrome, IR and increased BMI, body fat percentage and WC. Furthermore, a few MR studies provided more evidence that BMI had causal effect on 25OHD deficiency (6, 25), which was also found in our mvMR study. For one thing, most people with obesity and overweight would have lower outdoor physical activity and would cover their body with more clothes, so they might not get sufficient sun exposure (36). For another, being sequestered in adipose tissue, the bioavailability of vitamin D could get reduced in obesity patients (37). Many published articles (38) suggested the association of vitamin D deficiency and IR. It cannot be ignored is that most existing studies emphasized vitamin D played a significant role in the pathogenesis of IR (32), but the pathological mechanism of IR effect on vitamin D is not yet clear. Meanwhile, obesity and IR (even in the absence of obesity) are common and important feature of PCOS (39, 40). The above may account for the phenomenon of 25OHD deficiency in PCOS patients and the reason why this phenomenon disappears after adjusting for obesity and IR in our study. Anyway, our study provides new evidence that the effects of PCOS on 25OHD might be accounted for by obesity and IR.

Seeing that observational design is susceptible to reverse causality and potential confounders, current results of clinical studies between PCOS and 25OHD may bias the true relationship. For instance, most of the included studies were lack of control of BMI and IR. Obesity and IR have been confirmed to be important pathogenic factors of PCOS (41) and could lead to the decrease of 25OHD (6, 38, 42). Consequently, BMI and IR may be important mediators and confounders for the PCOS-25OHD relation, resulting that the independent PCOS-25OHD relation could not be assessed effectively. More importantly, no prospective large-scale longitudinal cohort studies have been conducted so far, it is still not sufficient to draw a clear conclusion on the causal relationship between PCOS and 25OHD. Unlike traditional observational epidemiological studies, our study reduced the impact of reverse causality and potential confounders, and explored the relationship between PCOS and 25OHD more accurately.

Nonetheless, several limitations in our study cannot be ignored. Firstly, the proportion of PCOS cases was relatively low and could bring compromised statistical power, failing to detect true causal relationship. Secondly, we failed to evaluate the causality between different clinical phenotypes of PCOS on 25OHD, due to the lack of GWAS data of PCOS phenotypes. We were not able to further stratify our outcomes and identify the risk of each phenotype, although there was great heterogeneity between different phenotypes of PCOS. Therefore, without further stratification, the causal relationship between PCOS and 25OHD obtained may be mixed and inaccurate. Thirdly, although many methods were used to control and evaluate the pleiotropy, the bias caused by gene pleiotropy could not be completely ruled out. Finally, the participants involved in our study were all from European ancestry. Therefore, it should take care when extending our conclusions to people of other ancestry.

## 5 Conclusion

In summary, our results highlighted that the causal effect of PCOS on 25OHD may be mediated by the negative effect of obesity and IR on 25OHD. Future studies with larger MR studies, clinical trials and further observational studies are highly warranted to confirm the results of our present study.

## Data availability statement

The original contributions presented in the study are included in the article/**Supplementary Material**. Further inquiries can be directed to the corresponding author.

## Author contributions

Conception and design: NZ; data curation: HZ and FJ; analysis: SZ and YY; software and visualization: CW and WZ; writing—original draft, review and editing: NZ and YL; funding acquisition: TC and XL. All authors contributed to the article and approved the submitted version.
